# Variation in Hounsfield unit calculated using dual-energy computed tomography: comparison of dual-layer, dual-source, and fast kilovoltage switching technique

**DOI:** 10.1007/s12194-024-00802-0

**Published:** 2024-05-03

**Authors:** Shingo Ohira, Junji Mochizuki, Tatsunori Niwa, Kazuyuki Endo, Masanari Minamitani, Hideomi Yamashita, Atsuto Katano, Toshikazu Imae, Teiji Nishio, Masahiko Koizumi, Keiichi Nakagawa

**Affiliations:** 1 Department of Comprehensive Radiation Oncology, The University of Tokyo, 7-3-1 Hongo, Bunkyo-ku, Tokyo 113-8655 Japan; 2 Department of Medical Physics and Engineering, Osaka University Graduate School of Medicine, Suita, Japan; 3 Department of Radiology, Minamino Cardiovascular Hospital, Tokyo, Japan; 4 Department of Radiology, Sakakibara Heart Institute, Tokyo, Japan; 5 Department of Radiologic Technology, Tokai University Hachioji Hospital, Tokyo, Japan; 6 Department of Radiology, The University of Tokyo Hospital, Tokyo, Japan

**Keywords:** Dual-energy CT, HU value, DLCT, DSCT, FKSCT

## Abstract

The purpose of the study is to investigate the variation in Hounsfield unit (HU) values calculated using dual-energy computed tomography (DECT) scanners. A tissue characterization phantom inserting 16 reference materials were scanned three times using DECT scanners [dual-layer CT (DLCT), dual-source CT (DSCT), and fast kilovoltage switching CT (FKSCT)] changing scanning conditions. The single-energy CT images (120 or 140 kVp), and virtual monochromatic images at 70 keV (VMI_70_) and 140 keV (VMI_140_) were reconstructed, and the HU values of each reference material were measured. The difference in HU values was larger when the phantom was scanned using the half dose with wrapping with rubber (strong beam-hardening effect) compared with the full dose without the rubber (reference condition), and the difference was larger as the electron density increased. For SECT, the difference in HU values against the reference condition measured by the DSCT (3.2 ± 5.0 HU) was significantly smaller (*p* < 0.05) than that using DLCT with 120 kVp (22.4 ± 23.8 HU), DLCT with 140 kVp (11.4 ± 12.8 HU), and FKSCT (13.4 ± 14.3 HU). The respective difference in HU values in the VMI_70_ and VMI_140_ measured using the DSCT (10.8 ± 17.1 and 3.5 ± 4.1 HU) and FKSCT (11.5 ± 21.8 and 5.5 ± 10.4 HU) were significantly smaller than those measured using the DLCT_120_ (23.1 ± 27.5 and 12.4 ± 9.4 HU) and DLCT_140_ (22.3 ± 28.6 and 13.1 ± 11.4 HU). The HU values and the susceptibility to beam-hardening effects varied widely depending on the DECT scanners.

## Introduction

In modern radiotherapy, computed tomography (CT), which is expressed as CT number (Hounsfield unit; HU), plays an important role in target delineation, treatment planning, patient positioning, and monitoring changes in tumor size [1–3]. In the treatment planning process, HU values are converted into the electron density, physical density, or stopping power ratio to simulate the interaction of radiation in the body [4, 5]. To understand the prescribed radiation dose for the target and surrounding organs at risk, the accurate calculation of HU values is imperative.

The X-ray used in the CT scanner contains a continuous energy spectrum with a mixture of low- and high-energies, and the low-energy photons are more likely to be absorbed by the body [6]. Therefore, the beam-hardening effect, which causes inaccurate calculation of HU value, is unavoidable, and the effect is caused by various factors such as scanning parameters, patient size, and the presence of high-density material such as bone [7]. Zurl et al. demonstrated that the variation in HU values caused the dose calculation error of 1.3% in the bone of the skull and 0.7% in the brain tissue [8].

Recently, dual-energy CT (DECT) increasingly introduced in clinical practice. The DECT can reconstruct various images such as material density images [9], and virtual monochromatic images (VMIs), and these images have been utilized for predicting treatment response and improving image quality [10]. The VMI at a given energy level (usually 40–140 keV) is reconstructed by acquiring data at two different X-ray energies, typically referred to as high and low energies. These energy levels can be achieved using different X-ray spectra or using different combinations of X-ray tube voltage and filtration. Typical beam hardening correction methods in single-energy CT (SECT) cannot fully remove the artifacts caused by the use of polychromatic X-ray spectra, whereas the VMI produced by DECT are principally less affected by the beam-hardening effect, resulting in the accurate HU calculation. The accurate calculation of HU values has the potential to improve dose calculation accuracy in the treatment planning process [11–13]. Moreover, Matsumoto et al. reported that the VMI at 70 keV provided better image quality (lower image noise and higher contrast to noise ratio) and could replace SECT (120 kVp) as the standard imaging method [14]. There are clinically available several types of DECT techniques: dual-layer CT (DLCT) [15], dual-source CT (DSCT) [16], fast kilovoltage switching CT (FKSCT) [17], twin-beam CT [18], and sequential scanning method [19]. However, DECT is not yet widely used in radiation therapy.

This study aims to investigate the variation in HU values measured using three types of DECT scanners (DLCT, DSCT, and FKSCT). Because DLCT can retrospectively reconstruct images such as VMI if conventional SECT scan is performed, it can be introduced without major changes to the CT imaging flow. DSCT uses two X-ray tubes, which allows for large changes in the energy spectrum of high- and low-energy X-rays, but the field of view is limited. FSKCT can acquire high- and low-energy projection data at approximately the same location, but the current value cannot be adjusted in detail. In addition, the impact of scanning conditions on HU value measurements using each of these devices is evaluated for introducing DECT in radiotherapy.

## Materials and methods

### Phantom and DECT scanner

This study does not require Institutional Review Board approval because only phantom is used. A tissue characterization phantom (model 467; Gammex RMI, Middleton, WI, USA) was utilized for all DECT scans, and the specification of tissue-mimicking reference material is shown in Table 1. The reference materials were inserted following the vendor-provided recommendation to minimize the artifact from the high-density materials.

**Table 1:** Electron density relative to water for reference materials

Number #	Reference material	Electron density relative to water
1	LN-300 lung	0.290
2	LN-450 lung	0.434
3	AP6 adipose	0.927
4	BR-12 breast	0.961
5	Water insert	1.000
6	CT solid water 1	0.989
7	CT solid water 2	0.989
8	CT solid water 3	0.989
9	CT solid water 4	0.989
10	BRN-SR2 brain	1.047
11	LV1 liver	1.061
12	IB inner bone	1.092
13	B200 bone mineral	1.106
14	CB2–30% CaCO3	1.277
15	CB2–50% CaCO3	1.469
16	SB3 cortical bone	1.683

The phantom was scanned using the following three types of DECT scanners. The DLCT (IQon spectral CT; Philips Healthcare, Amsterdam, Netherlands) equipped single-source, while divided the detector into two layers (yttrium-based garnet scintillator and gadolinium–oxysulphide). The low-energy photons are absorbed by the top layer and the high energy are by the bottom layer, and the VMIs were reconstructed based on these raw data sets. The DSCT (SOMATOM Drive; Siemens Healthineers, Forchheim, Germany) was equipped with dual-source and dual-detector, and the angular offset of both X-ray tubes was approximately 90°. In this scanner, the VMI was reconstructed based on two CT images acquired using each measurement system with different photon energies. The FKSCT (Aquilion ONE; Cannon Medical Systems, Otawara, Japan) switches between high- and low-energy during gantry rotation, and the VMIs are reconstructed based on two different raw projection data at almost the same gantry position.

### Image acquisitions

**Fig. 1:**
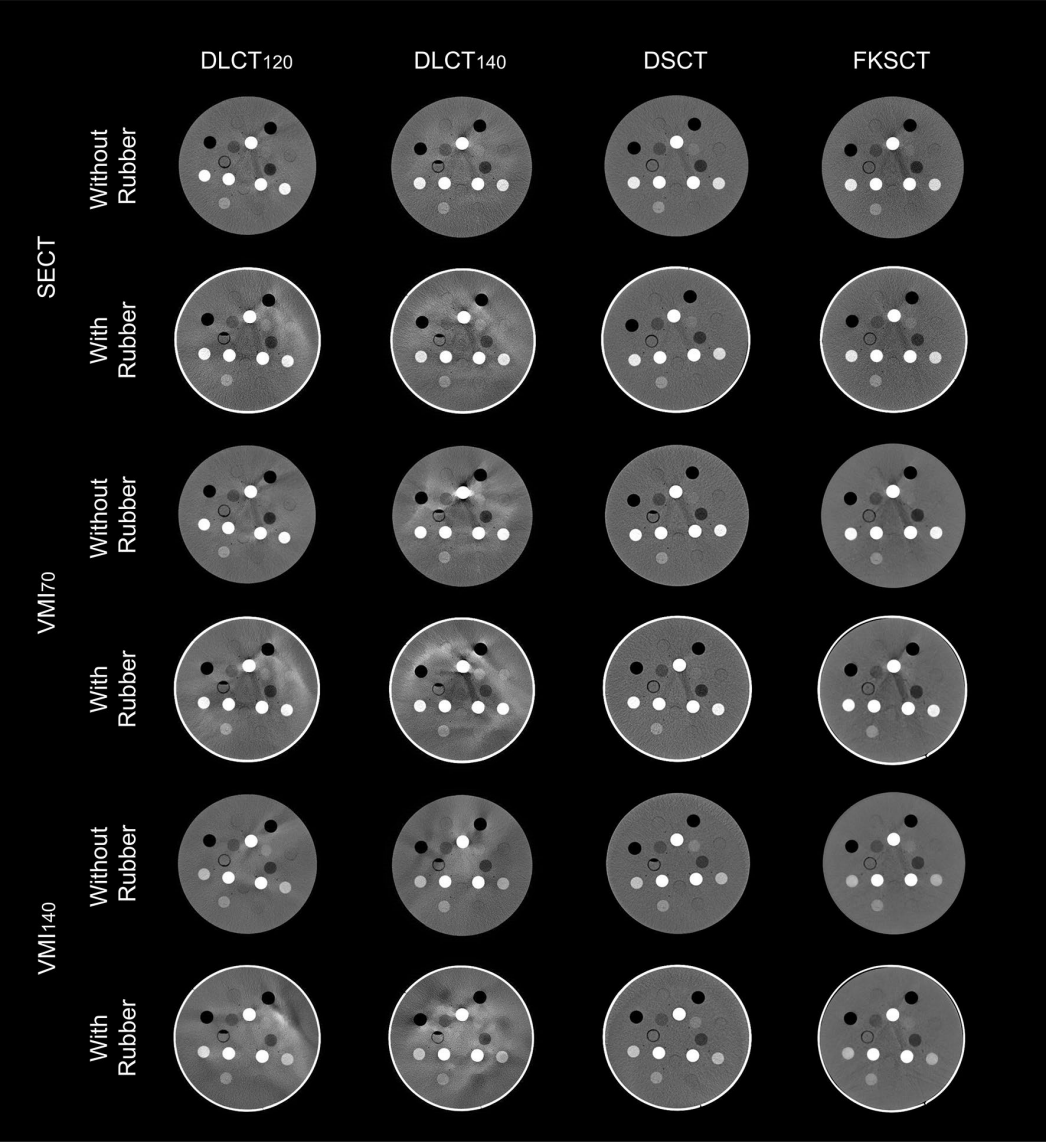
Single-energy computed tomography (SECT) images and virtual monochromatic images at 70 keV (VMI_70_) and 140 keV (VMI_140_) acquired using dual-layer CT with tube voltage of 120 (DLCT120) and 140 (DLCT140) kVp, dual-source CT (DSCT), and fast kilovoltage switching CT (FSKCT) scanners

The phantom was placed at the center of DECT scanners installed in the diagnostic department and was scanned three times to reduce random errors (Fig. 1). Table 2 shows the scanning parameters. For each DECT scanner, the volume CT dose index (CTDI_vol_) of approximately 25 mGy (full dose, protocol number #1, 3, 5, 7, 9, and 11) assuming a pelvic region (Gammex phantom is 33 cm in diameter) and approximately 12 mGy (half dose, protocol number #2, 4, 6, 8, 10, and 12) was used because CTDI_vol_ affects the accuracy of HU value calculation [20]. For other parameters except for the CTDI_vol_, an experienced radiation technologist for each device selected values similar to those used in clinical practice. Furthermore, the rubber-wrapped phantom was scanned to induce stronger beam-hardening effects. When scanning both hands down for such as esophageal cancer patients or patients with extra-large bodies, there is a risk of inducing beam hardening, resulting in HU values that are different from the original values. Therefore, if such beam hardening occurs, the electron density conversion table determined by each hospital may not function properly. Therefore, in this study, it was necessary to compare the accuracy of HU in each DECT with and without beam hardening induced. For DSCT and FSKCT scanners, the conventional SECT scan was also performed using the tube voltage of 120 kVp, and the VMIs at 70 keV (VMI_70_) and 140 keV (VMI_140_) were reconstructed for the DECT scan. The VMI at 70 keV is considered as the similar image quality with the conventional SECT image (120 kVp) [21, 22]. For the DLCT scan, the tube voltage of 120 (DLCT_120_) and 140 kVp (DLCT_140_) was used, and the SECT, VMI_70_, and VMI_140_ were simultaneously reconstructed at the same scan.

**Table 2:** Scanning parameters

Protocol number #	Scan mode	Type of DECT	Rows	Tube voltage (kVp)	Tube current (mA)	Rotation time (s)	Beam width (mm)	CTDIvol (mGy)	Pitch	DFOV (mm)	Slice thickness (mm)	Matrix size	Iterative reconstruction
1	DECT	DLCT	128	120	294	0.75	40	25	0.797	500	2	512	Level 0
2		DLCT	128	120	148	0.75	40	12	0.797	500	2	512	Level 0
3		DLCT	128	140	205	0.75	40	25	0.797	500	2	512	Level 0
4		DLCT	128	140	102	0.75	40	12	0.797	500	2	512	Level 0
5		DSCT	128	80/140	347/268	1	19.2	24.91	0.9	332	2	512	None
6		DSCT	128	80/140	170/132	1	19.2	12.1	0.9	332	2	512	None
7		FSKCT	320	80/140	580	0.5	40	24.2	0.813	400	2	512	Body mild
8		FSKCT	320	80/140	340	0.5	40	11.9	0.813	400	2	512	Body mild
9	SECT	DSCT	128	120	340	0.5	19.2	24.4	0.9	500	2	512	None
10		DSCT	128	120	170	0.5	19.2	12.2	0.9	500	2	512	None
11		FSKCT	320	120	570	0.5	40	24	0.813	500	2	512	None
12		FSKCT	320	120	300	0.5	40	12.1	0.813	500	2	512	None

The circular region of interest (ROI) was placed as large as possible, avoiding the boundary of the reference material, and the mean of HU values of three measurements was calculated for each material. Subsequently, the mean difference between the measured HU values with full dose and no rubber and the measured values when other scan parameters were used was calculated. The absolute difference in each reference material among DECT scanners was compared using the Kruskal–Wallis test to determine statistical significance. If significant differences were found, the Mann–Whitney *U* test was used to determine significant differences between DECT scanners. All statistical analysis was performed by SPSS software (IBM, Armonk, NY, USA), and a *p* value < 0.05 was considered to indicate statistical significance.

## Results

**Fig. 2:**
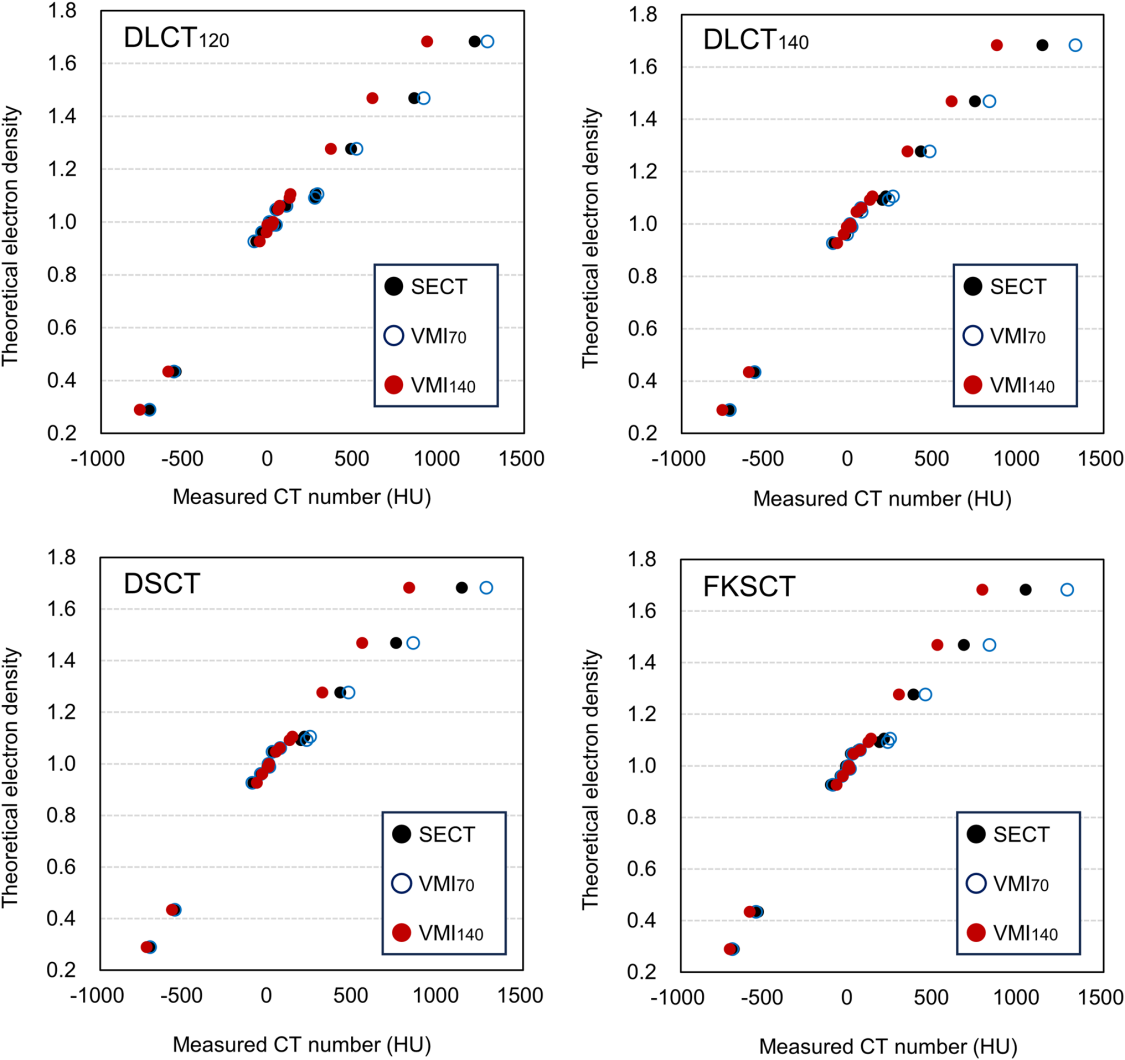
Relationship between measured HU values using DECT scanners and theoretical electron density relative to water when the phantom was scanned using the full dose without rubber. Error bars are too small to display

Figure 2 shows the relationship between measured HU values using DECT scanners and theoretical electron density relative to water when the phantom was scanned using the full dose without rubber. For each DECT scanner, VMI at 140 keV provided a linear relationship, while VMI at 70 keV formed bilinear relationships between measured HU values and theoretical electron density clustering around 0 HU. The difference in HU values between the SECT, VMI_70_, and VMI_140_ increased as the electron density of the reference material increased. For SB3 Cortical Bone, the mean HU value in the VMI_140_ was 925.9 ± 23.5, 867.5 ± 30.4, 826.4 ± 1.4, and 782.6 ± 4.4 HU for DLCT_120_, DLCT_140_, DSCT, FSKCT, respectively, and that in the VMI_70_ was 1282.0 ± 10.5, 1333.5 ± 18.4, 1283.1 ± 3.0, and 1285.6 ± 13.3 HU, respectively.

**Fig. 3:**
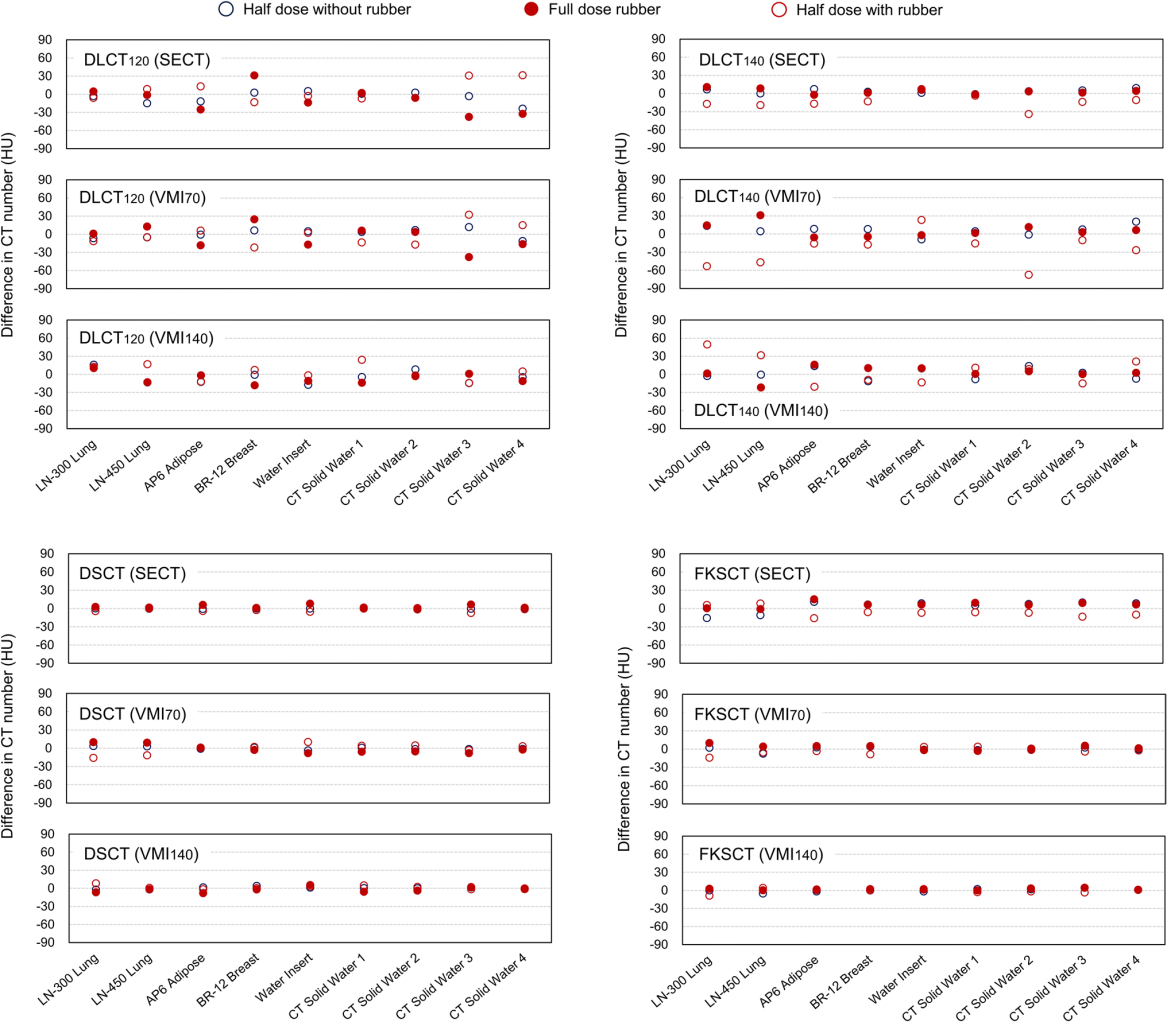
Difference in measured HU values between the full dose scan without rubber and the other scanning conditions for low-density materials

**Fig. 4:**
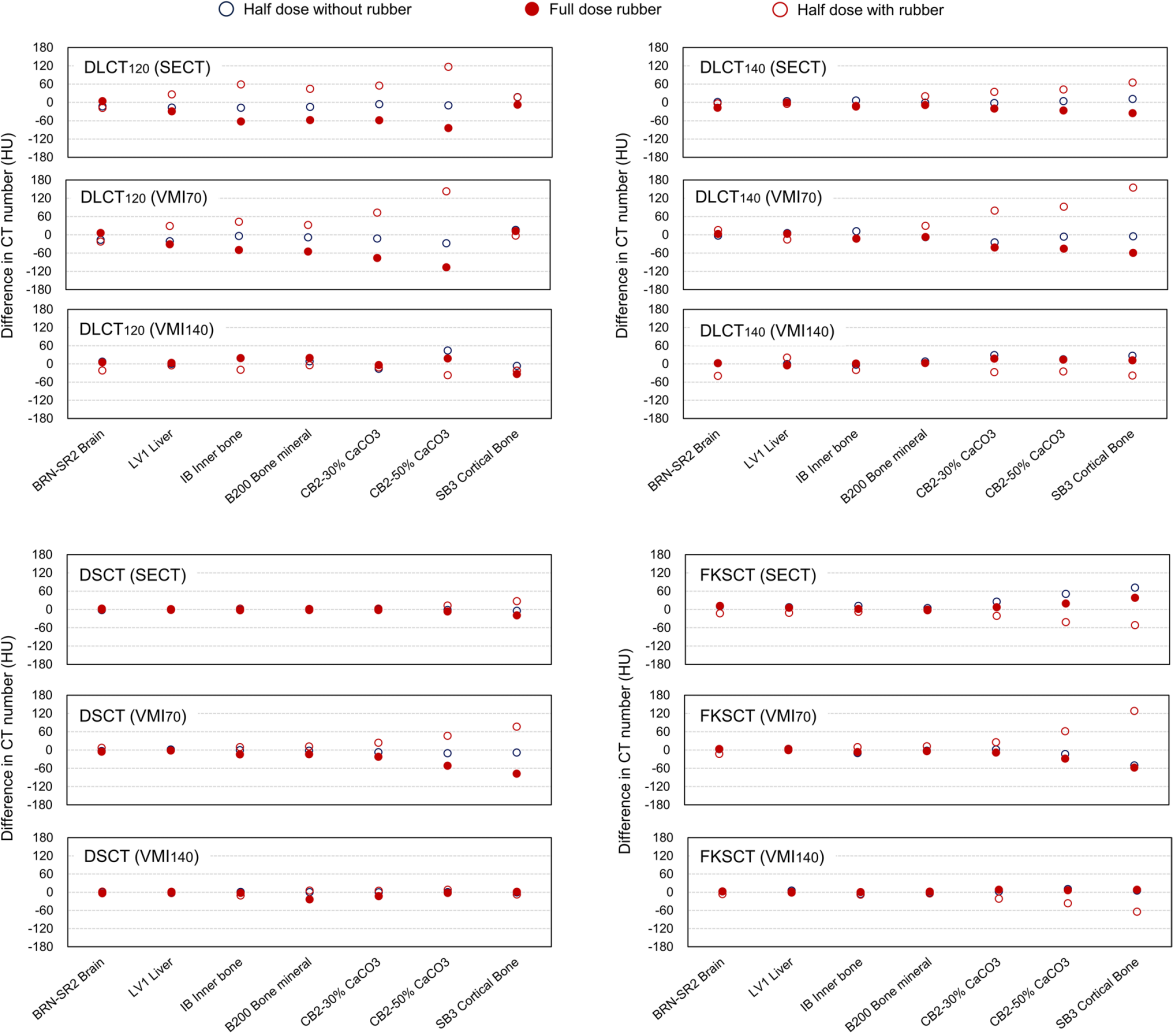
Difference in measured HU values between the full dose scan without a rubber and the other scanning conditions for high-density materials

Figures 3 and 4 show the mean difference in measured HU values between the full dose scan without rubber and the other scanning conditions for low-density (reference material number #1–9) and high-density materials (reference material number #10–16), respectively. Overall, the mean difference in HU values was larger when the phantom was scanned using the half dose with the rubber, and the mean difference was larger as the electron density increased. For SB3 Cortical Bone, the maximum mean difference of 155.2 HU was observed in the VMI_70_ acquired using DLCT_140_ with the half dose with the rubber (Fig. 4).

**Fig. 5:**
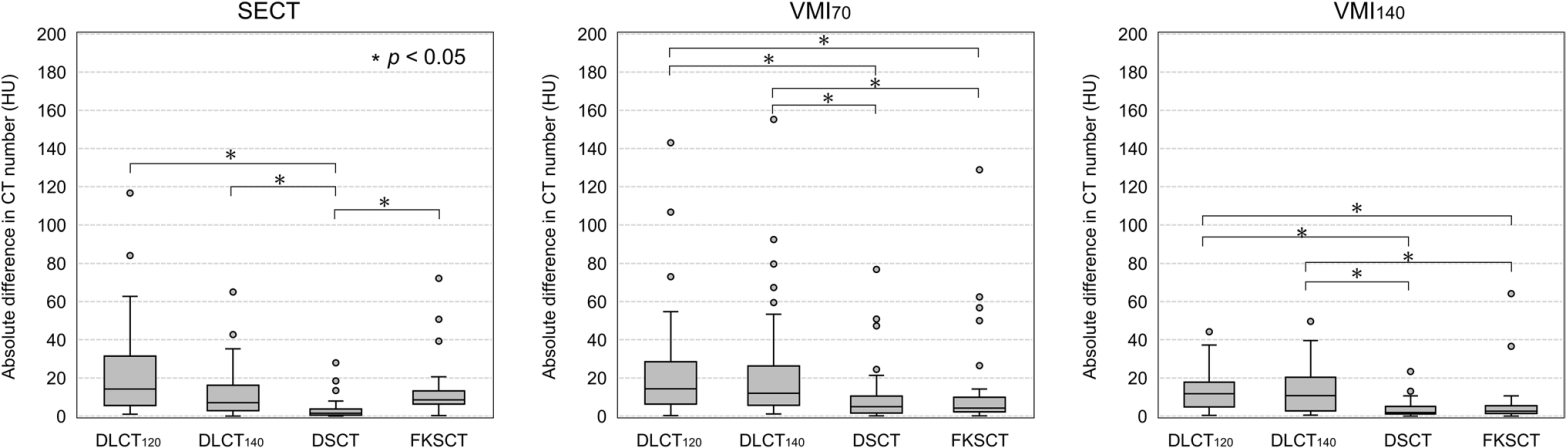
Comparison of absolute differences in measured HU values between the full dose the full dose scan without rubber and the other scanning conditions

Figure 5 shows the comparison of absolute differences among DECT scanners in measured HU values between the full dose scan without rubber and the other scanning conditions. For SECT, the difference in HU values measured by the DSCT (3.2 ± 5.0 HU) was significantly smaller (*p* < 0.05) than that using DLCT_120_ (22.4 ± 23.8 HU), DLCT_140_ (11.4 ± 12.8 HU), and FKSCT (13.4 ± 14.3 HU). The respective difference in HU values in the VMI_70_ and VMI_140_ measured using the DSCT (10.8 ± 17.1 and 3.5 ± 4.1 HU) and FKSCT (11.5 ± 21.8 and 5.5 ± 10.4 HU) were significantly smaller (*p* < 0.05) than those measured using the DLCT_120_ (23.1 ± 27.5 and 12.4 ± 9.4 HU) and DLCT_140_ (22.3 ± 28.6 and 13.1 ± 11.4 HU).

## Discussion

In this study, we investigated the variation in HU values of the tissue characterization phantom calculated using DECT scanners. The HU value of a given material is calculated as: HU = (*μ*_material_–*μ*_water_)/*μ*_water_ × *1000*, where *μ*_material_ and *μ*_water_ indicate the linear attenuation coefficient for a given material and water, respectively. Because the linear attenuation coefficient varies with X-ray energy levels, the HU values also vary depending on the energy used even for the same material. Cropp et al. demonstrated that the measured HU values, especially for high-density materials differed among SECT models and manufacturers even if the same nominal tube voltage was used [23]. The reason for this is due to differences in the X-ray energy spectrum caused by differences in the target material, X-ray tube, and other structures for each SECT scanner. The energy spectrum is also affected by the environment surrounding a given material (phantom size and presence of dense material), and thus, accurate measurement of HU values is difficult with SECT.

Theoretically, VMIs derived from the DECT scanners yield a more accurate calculation of HU by eliminating the beam-hardening effect, and ideally, the HU value in the VMI would be the same regardless of which type of DECT scanner is used. However, Chen et al. examined the accuracy of HU estimation among multivendor DECT scanners and found that the mean absolute percentage error between measured and theoretical HU values varied depending on the types of DECT acquisition techniques and the generation of the model even for the same vendor [24]. In the report, the latest model of FKSCT (Revolution Apex, GE Medical Systems, Milwaukee, WI, USA), and DSCT (SOMATOM Force; Siemens Healthineers) scanners provided more accurate HU values compared with the DLCT scanner. In the DLCT, the detector distinguishes between high- and low-energy X-rays, but since only one X-ray energy is used, the VMI is reconstructed using X-rays affected by beam hardening, which is thought to have caused the larger error. In contrast, the smaller differences for SB3 in DLCT_120_ was observed (Fig. 4), and this might be because the error due to the uncertainty of the measurements cancels out the error due to the streak artifacts as shown in Fig. 1. In radiotherapy, robustness is considered more important than the error of the HU value from the theoretical value, because the conversion table between HU value and electron density or material density is generated for the dose calculation. Therefore, the importance of our study is the evaluation of the robustness of the HU values under conditions that strongly induce beam hardening effects (rubber wrapping). Zurl et al. demonstrated that a coarse HU estimation for the skull bone can cause the uncertainty of dose calculation with ∆ dose (%) = 0.15 × ∆HU (%) [8]. Therefore, the difference of 100 HU in dense material can cause dose errors of about 1%. Previous investigators demonstrated that the VMIs at lower energy (63 keV) could increase the HU value of contrast-enhanced agent and improves objective and subjective image quality compared with the SECT images [25]. Although the higher image quality may reduce the variability of target delineation depending on the radiation oncologist, but the accuracy of HU calculation might be degraded [7]. Care should be taken when performing dose calculations based on low-energy VMI.

In this study, VMI_140_ was the least affected by different scanning conditions in the calculation of HU values across all DECT scanners, and previous studies have reported that VMI at high energy levels may improve the accuracy of dose calculation in radiation therapy planning [26, 27]. The VMI at a high energy level not only improves the robustness of HU value calculations but also reduces metal artifacts that frequently reduce the accuracy of dose calculations in the treatment planning process [28]. Further, the contrast-enhancement agent-induced increase in HU values decreases as VMI energy increases, and thus, the VMI at a higher energy level has the potential to ensure dose calculation accuracy in treatment planning based on contrast-enhanced CT [29]. However, the VMI at a high energy level results in low contrast resolution between soft tissue and bone, therefore careful consideration should be given to its use for target contouring. The beam-hardening effects cannot be completely eliminated even at high-energy VMI. Recently, Ji et al. developed physics-driven deep learning-based method, and their proposed method achieved the better performance in both qualitative and quantitative aspects compared with the conventional beam-hardening correction method [30]. The newly developed reconstruction technique has the potential to reduce the beam hardening effect resulting in accurate HU calculation.

This study includes several limitations. First, although only HU values were considered in this study, it has been reported that dose calculations based on effective atomic number and electron density images are more accurate than HU values for particle therapy [31]. Second, there are numerous variations of scanning parameters (tube current, gantry rotation speed, helical pitch, reconstruction filter, slice thickness, and so on) that were not all investigated in this study. Third, the DLCT scanner was most affected by differences in scanning conditions in this study, but the latest models of DLCTs (Spectral CT 7500, Philips Healthcare) were not used. Fourth, this study was conducted on phantoms only, and it is not assured that the same results would be achieved in the human body. Finally, even if the same model of DECT scanner is used, the measurement results may vary from one device to another.

In conclusion, the HU values in the SECT, VMI_70,_ and VMI_140_ acquired using the DLCT, DSCT, and FKSCT varied even for the same tissue characterization phantom. Across all DECT scanners, the VMI_140_ showed the least variation in HU value calculations under different scanning conditions. The robustness of DSCT and FKSCT by scan condition may improve the accuracy of dose calculations in radiotherapy.

## Data Availability

Data will be made available on reasonable request.
